# Energy crypto currencies and leading U.S. energy stock prices: are Fibonacci retracements profitable?

**DOI:** 10.1186/s40854-021-00311-8

**Published:** 2022-01-12

**Authors:** Ikhlaas Gurrib, Mohammad Nourani, Rajesh Kumar Bhaskaran

**Affiliations:** 1 Faculty of Management, Canadian University Dubai, 1st Interchange, Sheikh Zayed Road, P.O. Box 117781, Dubai, United Arab Emirates; 2 The University of Waikato Joint Institute at Zhejiang University City College, University of Waikato, Hangzhou, China; 3 Department of Finance, Institute of Management Technology, UG 02, Dubai International Academic City, Dubai, United Arab Emirates

**Keywords:** Performance evaluation, Energy cryptos, Energy stocks, Fibonacci retracements

## Abstract

This paper investigates the role of Fibonacci retracements levels, a popular technical analysis indicator, in predicting stock prices of leading U.S. energy companies and energy cryptocurrencies. The study methodology focuses on applying Fibonacci retracements as a system compared with the buy-and-hold strategy. Daily crypto and stock prices were obtained from the Standard & Poor's composite 1500 energy index and CoinMarketCap between November 2017 and January 2020. This study also examined if the combined Fibonacci retracements and the price crossover strategy result in a higher return per unit of risk. Our findings revealed that Fibonacci retracement captures energy stock price changes better than cryptos. Furthermore, most price violations were frequent during price falls compared to price increases, supporting that the Fibonacci instrument does not capture price movements during up and downtrends, respectively. Also, fewer consecutive retracement breaks were observed when the price violations were examined 3 days before the current break. Furthermore, the Fibonacci-based strategy resulted in higher returns relative to the naïve buy-and-hold model. Finally, complementing Fibonacci with the price cross strategy did not improve the results and led to fewer or no trades for some constituents. This study’s overall findings elucidate that, despite significant drops in oil prices, speculators (traders) can implement profitable strategies when using technical analysis indicators, like the Fibonacci retracement tool, with or without price crossover rules.

## Introduction

**Fig. 1:**
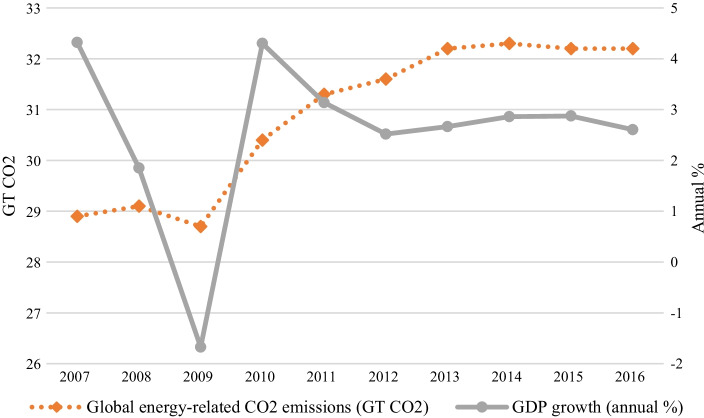
The world’s GHG emissions (energy-related CO_2_ emissions) and GDP growth from 2007 to 2016. *Source*: Authors’ estimate based on IEA CO_2_ emissions from burning of fuel (https://iea.org/subscribe-to-data-services/co2-emissions-statistics), World Bank national accounts data, and OECD national accounts data files

Decoupling, decarbonization, and energy policy are buzzwords hitting major headline discussions on the global energy market, particularly in the United States. Energy markets have usually been linked with Gross Domestic Product (GDP) growth, as energy trades in oil and gas are critical components of the global commodity trade (Zhang 2019). The International Energy Agency (IEA) observed that, although in 2016 there was continuous GDP growth at around 3% annually, the world’s greenhouse gas (GHG) emissions remained constant from 2014 to 2015 (IEA 2016, 2015). The revelation was encouraging, as the GHG and global growth finally decoupled, eventually leading to a less than 2 °C increase in global average surface temperature from pre-industrial levels (UFCCC 2016; Chemnick 2016). Figure 1 reiterates and illustrates the decoupling of GHG emissions and GDP growth in the world between 2007 and 2016.

**Fig. 2:**
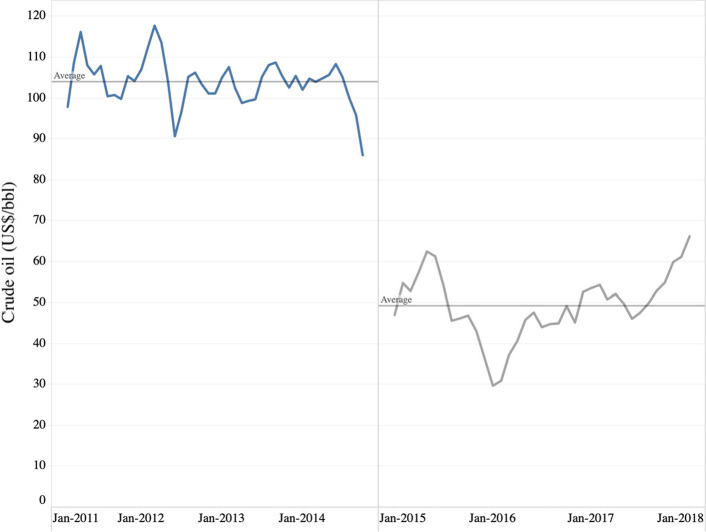
Average monthly crude oil price in nominal US dollars from 2011 to 2018. *Source*: Authors’ estimate based on the average of Brent, Dubai, and WTI data provided by World Bank

However, during 2014–2018, as shown in Fig. 2, oil prices dropped by more than 67%, with current prices hovering around 45% of the 2011–2014 values. Consequently, several oil-reliant economies were affected by significant reductions in consumption and investment progress (World Bank 2018). Such changes in energy prices led to risk in economic activities (Jibril et al. 2020), forcing various countries to use different adequate state policies and procedures to rely less on oil. In the same vein, this supports that investors will be more careful when pursuing investment activities related to commodity and equity markets, led by the crude oil market (Jiang et al. 2020). Although globalization promotes dependence across markets, such relationships are not forthright, especially with emerging alternative financial products (Qarni and Gulzar 2021). For example, Gurrib (2019) found that the price indices of energy commodities and cryptocurrencies were not strong predictors of energy cryptocurrency and energy commodities. Gurrib and Kamalov (2019) found that the reward to volatility ratio changed in crude oil and natural gas before and after the 2008 global financial crisis. However, Gurrib (2018a) reported that using an index constructed from most used fossil fuels could not forecast key equity market indices movements during the 2000 technology crisis. Similarly, Gupta et al. (2017) found that risk futures markets’ volatility rose gradually and are unrelated to other financial markets’ volatilities.

The energy market is constantly evolving; EIA (2018) forecasts a higher energy consumption of the electric power sector than any other sector and further importance of renewable energy consumption compared to other energy sources. Natural gas consumption is also predicted to rise due to the booming industrial sector, especially power, heat, and liquid natural gas production. Although natural gas production is forecasted to represent almost 40% of the U.S. energy production within 30 years, wind and solar power use currently lead, compared to other renewable energies. Increasingly, power plants using fossil fuels are being substituted with solar panels and microturbines. Several governments have become more conscious of global climatic conditions; with more subsidies for cleaner energies and decreasing wind and solar power charges, renewable energies are predicted to provide more than 10% of the global electricity supply between 2017 and 2022 (EIA 2018).

Whether at spot or futures, crude oil prices impact commodities and alternative asset classes, like stocks (Kirikkaleli and Güngör 2021). Faced with challenging events, such as the Middle East sanctions, China trade wars, COVID-19 pandemic, decoupling of energy commodities, and cryptocurrencies, energy policymakers, such as the Commodity Futures Trading Commission, are working vehemently to ensure market depth and liquidity while overseeing price volatility. The drop in energy stock prices from July 2014 to December 2015 due to the oil price slump provides a good reference point. Additionally, the decline of energy cryptocurrencies after December 2017 is another significant instance to mention. Among others, traders use technical and fundamental tools to derive profits through some set trading methodologies.

While different trading approaches the proof of market success, such as currencies, stock and bond, and cryptocurrency markets (Nadarajah and Chu 2017; Neely et al. 2014; Shynkevich 2012, 2016), financial market uncertainties make technical and fundamental techniques more challenging for investors or traders to utilize. Pivotal research on the value of technical analysis can be linked back to Ball (1978) and Fama (1970). The former study found that market-timing strategies led to negative returns following the adjustment for transaction charges. The latter study backs the Efficient Market Hypothesis that actual market prices represent all information available currently, such that relying on those may not be profitable or result in a positive return accompanied by an undesirable level of risk. The results of Fama and Ball were supported by Park and Irwin (2010), who argued that rules of technical analysis did not yield consistent gains in U.S. futures. Pruitt and White (1988), however, concluded that their technology-based system, which contained relative strength index (RSI), volume, and moving average (MA), was superior to the market after adjusting for transaction charges. Similarly, Menkhoff (2010) discovered that most country fund managers adopted technical analysis in various countries. To support technical analysis further, Szakmary et al. (2010) reported strategies based on trends to be profitable in commodity futures, while Tsaih et al. (1998) noted that their trading system was superior to the use of a buy-and-hold trading rule for Standard & Poor's (S&P) 500 futures. Wong et al. (2003) observed that using MA and RSI and MA result in substantial gains in the Singapore Stock Exchange. Also, Neely et al. (2009) detected that, when using technical analysis, profitability and market conditions change as time passes. This supports Gurrib (2018b), who used the Average Directional Index (ADX) for currencies paired against the U.S. dollar, and reported that relying on weekly horizons, compared to monthly, yielded more profits. Beyaz et al. (2018) studied several companies using technical and fundamental approaches. They discovered varying performance in both; utilizing either mechanism was less pronounced for energy equities, while the combination of both tools yielded better equity price predictions. Loginov et al. (2015) compared the use of Fibonacci retracements with MA and pivot points, and found that Fibonacci retracements yielded better results in the foreign currency market. Although previous studies tend to include various technical analysis tools, the application of Fibonacci retracements on energy stocks, and more importantly on energy cryptos, is rare. To the best of the author’s knowledge, no study has investigated whether a price crossover strategy coupled with Fibonacci retracements can yield a superior trading system. To meet this study’s objective, we evaluated the performance of Fibonacci retracements as a trading system and provide a comparison with the naïve buy-and-hold model.

Based on the top-ten energy equities, this analysis is the first to provide some insight into whether there is some cohesion in the performance of energy-based companies when using Fibonacci retracements. This study adds to the existing literature on financial innovation in two ways. First, it compares the results of the Fibonacci retracement trading strategy with the buy-and-hold strategy and assists in answering whether Fibonacci retracements are more reliable. The performance is captured using the Sharpe and Sharpe per trade performance measure and subsequently compared with the conventional buy-and-hold strategy, thereby guiding the best technical analysis tools to predict energy stock prices. Second, the study examines whether including a price crossover strategy with the Fibonacci trading system results in a higher return per unit of risk. Our findings support that Fibonacci retracements can be incorporated into a trading strategy with significant returns for energy sector stocks compared to cryptocurrencies. The study suggests that price violations are observed more during downtrends than uptrends. In the context of return generation, the Fibonacci strategy is superior to the naïve buy-and-hold model. Complementing the Fibonacci retracement strategy with the price crossover strategy is not an effective trading model for energy-based commodities.

The policy implications are also laid out in terms of whether disruption in commodity prices, like drops in oil prices, affect the profit potentials of traders’ techniques or, more specifically, speculators in energy markets trades. The rest of the paper presents the literature review of the performance measure used, the descriptive statistics of the data, the methodology applied in setting the trading system, the research findings, and finally, the concluding remarks.

## Literature review

A substantial amount of literature is available on technical analysis and financial markets. For instance, Smith et al. (2016) reported that 20% of hedge funds used technical analysis. Kamalov et al. (2021) forecasted the direction of U.S. large-cap stocks and found that adding technical indicators equalized the effectiveness of return and price as inputs in machine learning models. Gencay (1999) found gains in foreign currency markets, with Olson (2004) further supporting that risk-adjusted trading rule gains gradually fell as time passed. Brock et al. (1992) similarly found that technical trading methodologies led to significant predictions for the Dow Jones Industrial Average (DJIA) over 90 years. Psaradellis et al. (2019) used over seven thousand trading rules and reported temporary profitable trading opportunities only in crude oil futures. The same author’s findings are also supported by adaptive market hypothesis proponents, such as Lo (2019) and Urquhart et al. (2015). They believed that markets and investors adapt, suggesting that technical trading systems tend to gradually lose their forecasting power.

There is abundant literature on technical analysis usage in several markets, like foreign currencies. However, applications in energy markets have been covered relatively more recently because of oil financialization, making oil-based contracts an attractive financial product for experienced traders of crude oil futures (Zhang 2017; Creti and Nguyen 2015). Although there is limited literature on the association between technical analysis and energy equities markets, the connection represents a reference point for potential relationships. Marshall et al. (2008a) applied seven thousand rules on key commodity futures and reported that only a few strategies resulted in consistent gains after allowing for data snooping adjustments. Contrary to this finding, Szakmary et al. (2010) found that MA strategies yielded positive returns for most commodity futures. Narayan et al. (2015) similarly support that momentum-based trading strategies can be profitable by taking long (short) positions in the best (worst) performing commodities. Similarly, Narayan et al. (2013) reported that trading strategies using Simple MA (SMA) yielded noteworthy gold and oil commodities returns. Although the same authors also reported that oil commodity futures could forecast returns in the spot market, Gurrib (2018a) found an energy index unreliable in predicting major equity market indices; this finding was backed by Aggarwal (1988), who supported an increase in volatility both after the futures markets were initially introduced and later, as time passed; thus, confirming that futures markets are not inevitably linked to other market volatilities. Therefore, additional factors, like uncertainty, may be responsible for volatility in markets.

Lately, Czudaj (2019) adopted technical tools for momentum trading in crude oil and reported that responses to unexpected events significantly fluctuated when assessed over different frequency periods. High (low) frequencies were accompanied by a temporary (persistent) response to uncertainty shocks. Furthermore, Marshall et al. (2008b) found that investors depend more on technical analysis tools for predictions over the short run and stress that technical indicators were used more for intraday trading relative to yearly trading horizons. In addition to confirming the application of Fibonacci retracements to derive returns, our analysis further adds to the existing financial innovation literature by comparing the findings with the naïve strategy. This study also taps into whether complementing the Fibonacci retracement with the price crossover strategy improves the profitable opportunities of energy stocks and energy cryptos.

Prices of financial products are known to increase, decrease, and pause for consolidation, and occasionally retrace before resuming onwards evolution. The performance of the S&P 500 is a good example, showing two major global crises in 2000 and 2008 before resuming its uptrend from 2009 to 2020. Many finance practitioners have long believed, and continue to assume, that these retracements can be predicted through the various Fibonacci series propositions (Posamentier and Lehmann 2007). The use of Fibonacci can be found in automated trading systems, such as harmonic trading, and specific harmonic price patterns to define highly probable reversal points in financial products’ prices. Such patterns can be identified, and positions can be taken based on the belief that historical price movements will be similar. Hurst (1973) reported that the periods of neighboring waves in price movements tend to be related by a small whole number, which Fibonacci retracement levels can probably determine. Harmonic price patterns, which are based on the Elliott wave theory (see Elliott 1935), and Fibonacci are conceptually similar^1^ owing to their assumed correction of prices at some point. However, it is important to note that the Fibonacci tool necessitates specific retracement levels aligned to the Fibonacci or conjugate golden ratio. Although there is abundant coverage of the Fibonacci tool in the extant literature (Bhattacharya and Kumar 2006), its use in the energy sector is relatively scarce.

Otake and Fallou (2013) analyzed the use of the Fibonacci ratios in the African regional stock change and reported the tool to help predict retracements. Similarly, Lahutta (2016) found similar usefulness when applied over the Warsaw stock exchange. Gartley (1935) introduced the Gartley pattern, positing that any retracement pattern must first be initiated with a 61.8% retracement (the conjugate golden ratio). He found it to be one of the most profitable strategies for the stock market.

After surveying foreign currency dealers in Hong Kong, Lui and Mole (1998) reported that technical analysis is less significant in forecasting trends than fundamental analysis but ominously more beneficial to forecast turning points in prices. More essentially, MA trend-following systems and moving averages were the most rewarding techniques. Such trading rules are more commonly adopted because people adjust less by staying close to their anchors (referring to the investment tools often adopted), as Epley and Gilovich (2006) proposed. They suggested that alteration to other techniques is indeed a task requiring significant effort. Although the literature on the enhanced value of trend-following systems is plentiful, Zweig (2009) and Hayes (2001) provided a worthy overview of early systems, such as the Dow Theory, upon which the current DJIA is constructed.

With particular reference to the MA, technical analysis systems can be linked back to Cowles (1933) and Tintner (1935); possibly, the most quoted long-term trend measurement is the 200 days MA. Siegel (2014) tested the long-run MA on the DJIA and the Nasdaq composite index over the 1886–⁠2006 period and found the market-timing strategy outperformed the buy-and-hold strategy. Using a comparable method, Faber (2007) reported similar results for the 1901–⁠2012 period. Using an MA strategy had fewer large losses and gains instances, with congruently higher occurrences of small losses and gains. This suggests that the MA strategy tends to the far-left tail of big losses, though it sacrifices the far-right tail of big gains.

Gurrib (2016) proposed an MA strategy based on optimization parameters over the Standard & Poor's Depositary Receipt (SPDR) S&P 500 exchange-traded fund using a heating map. They reported that the market-timing strategy outperformed the naïve buy-and-hold strategy over 1993–⁠2014, with a relatively higher reward to volatility.

Performance measuring tools including Sharpe, M^2^, Jensen’s alpha, and Treynor are commonly adopted in portfolio management companies to capture the capacity of portfolios using market-timing tools. Asset pricing tools, aligned with the introduction of performance measuring tools, were introduced as a means to discover the portfolio components that should trigger higher or lower expected returns. For example, the Capital Asset Pricing Model conceptually presented in Sharpe (1964) assumes that market risk elements impact the portfolio. Although Jensen’s alpha (Jensen 1968) relies on the difference between expected and actual returns, it does not account for firm-specific risk, imperative to the investor (Fama 1972). Similarly, Treynor’s ratio, developed by Treynor (1965), contemplates only the excess return per unit of market risk, like Jensen’s alpha, as reviewed in Aragon and Ferson (2007). The reward to volatility ratio or Sharpe ratio, introduced by Sharpe (1964), represents the excess returns for each unit of risk; excess returns represent the difference between the risk-free rate and return. The former is usually proxied by the 3-month U.S. Treasury bill rate.

## Research methodology

### Data

We chose the top-ten energy companies from the S&P Composite 1500 Energy Index to meet the study's objectives. The index captures the performance of publicly listed companies that are members of the Global Industry Classification Standard energy sector. Launched on December 31, 2005, the index has 89 constituents with a maximum and mean market capitalization value of $314,624 million and $14,677 million, respectively, as of July 31, 2019. The leading ten stocks were chosen based on their relative weights in the index, represented in Table 1.

**Table 1:** Company specifications. *Source*: S&P 500 Dow Jones Indices, Factset

Company	Trading symbol	Sector	Industry	Sub industry
Exxon Mobil	XOM	Energy	Oil, Gas & Consumable Fuels	Oil & Gas Exploration & Production
Chevron Corp	CVX		Oil, Gas & Consumable Fuels	Integrated Oil & Gas
ConocoPhillips	COP		Oil, Gas & Consumable Fuels	Oil & Gas Exploration & Production
Schlumberger Ltd	SLB		Energy Equipment & Services	Oil & Gas Equipment & Services
EOG Resources	EOG		Oil, Gas & Consumable Fuels	Oil & Gas Exploration & Production
Occidental Petroleum	OXY		Oil, Gas & Consumable Fuels	Oil & Gas Exploration & Production
Marathon Petroleum Corp	MPC		Oil, Gas & Consumable Fuels	Oil & Gas Refining & Marketing
Phillips 66	PSX		Oil, Gas & Consumable Fuels	Oil & Gas Refining & Marketing
Anadarko Petroleum Corp	APC		Oil, Gas & Consumable Fuels	Oil & Gas Exploration & Production
Kinder Morgan Inc	KMI		Oil, Gas & Consumable Fuels	Oil & Gas Storage & Transportation

**Fig. 3:**
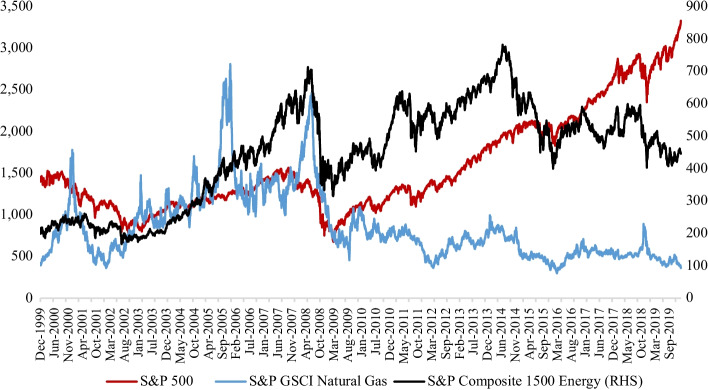
Performance of S&P 1500 Energy, S&P 500, and Natural Gas. *Note*: Displayed on the left side of this figure is the performance of S&P 500 index, while on the right side are those of S&P Composite 1500 Energy and S&P GSCI Natural gas market indices. The period covered is from December 1999 to July 2019. *Source*: S&P 500 Dow Jones Indices, Factset

It is vital to comprehend that the S&P Composite 1500 Energy index has been unpredictable relative to the S&P 500 and the S&P Goldman Sachs Commodity Index (GSCI) Natural Gas indices, thus providing investors with a benchmark of the natural gas market’s performance. Figure 3 provides a summary of the performance of the three market indices. From late 2008, the crude oil and natural gas markets decoupled. On the one hand, the need for oil to produce electricity has fallen vastly due to the gradual withdrawal of highly depreciated petroleum assets, falling natural gas prices, availability of better gas-fired engines, and increased awareness of the climatic consequence of oil’s high sulfur content. On the other hand, despite the growth of associated gas in the U.S., the largest producer of natural gas, robust supply coming from shale players, like Utica/Marcellus, has dampened the impact of the growth of natural gas prices (Mchich 2018). Post-2008, the S&P 500 performed comparatively better than the S&P 1500 composite energy index, shown in the left and right sides of Fig. 3. Fluctuations, seen in the S&P Composite 1500 Energy Index, position the Fibonacci retracement tool as a conceivable indicator for future adoptions in investment decision making; it is assumed that volatility encompasses retracements and expansions. To allow for the current (as of January 2020) top-ten energy stocks in the S&P Composite 1500 Energy Index to be analyzed, the study period was set between November 21, 2017, and January 17, 2020. The risk-free rate (annualized) of 1.20% was based on the 3-month U.S. Treasury bill rate, which varied from a minimum value of 1.25% to 2.43% during the study period. We obtained the rate from the St. Louis Federal Reserve database, energy crypto data from CoinMarketCap,^2^ and energy equity prices from Factset.

### Fibonacci retracements

During the Middle Ages, the mathematician Leonardo Fibonacci discovered that the Fibonacci numbers form a sequence of integers found in various entities ranging from nature (e.g., birth rates of rabbits) to mathematics (e.g., the Pascal triangle (Livio 2008)). The *n*th Fibonacci number is structured as follows:

1 $$\begin{aligned} \theta_{{\text{n}}} = 1, &\quad {\text{for}}\,{\text{n}} = 0,1 \\ \theta_{{\text{n}}} = \theta_{{{\text{n}} - 1}} + \theta_{{{\text{n}} - 2}} , &\quad {\text{for}}\,{\text{n}} \ge 2 \\ \end{aligned}$$

The Fibonacci recursive relationship model is based on the use of consecutive numbers from the Fibonacci series. Dividing both sides of Eq. (1) by ${\uptheta }_{{\text{n - 1}}}$, the following form is gathered:

2 $$\frac{{{\uptheta }_{{\text{n}}} }}{{{\uptheta }_{{{\text{n}} - 1}} }} = 1 + \frac{{{\uptheta }_{{{\text{n}} - 2}} }}{{{\uptheta }_{{{\text{n}} - 1}} }}$$

As ${ }n \to \infty$, $\frac{{\theta_{n} }}{{\theta_{n - 1} }} \approx \frac{{\theta_{n - 1} }}{{\theta_{n - 2} }}$. Substituting $\frac{{\theta_{n} }}{{\theta_{n - 1} }}{ }$ as α, Eq. (2) is reduced to:

3 $$\mathop {\lim }\limits_{{{\text{n}} \to \infty }} {\upalpha } = 1 + \frac{1}{{\upalpha }}$$

Solving for α from Eq. (3) for infinitely large values of *n*, the limiting value of the Fibonacci ratio can be obtained by solving for the roots of the polynomial ${\upalpha }^{2} - {\upalpha } - 1$. The larger of the two roots gives rise to what is dubbed as the golden ratio value of 1.618, while the lower value of roots creates the golden ratio conjugate, valued at 0.618. Meanwhile, the golden ratio value is the reciprocal of the golden ratio conjugate value. Although not detailed further here for brevity, some essential properties of the golden ratio include: (i) it is equal to its own reciprocal plus 1 (continued fractions); (ii) it is equal to its square root plus 1 (nested radicals); most importantly, (iii) it approaches the value of 1.618 as *n* increases; and (iv) its reciprocal, i.e., $\frac{{\theta_{n - 1} }}{{\theta_{n} }}$ approaches the value of 0.618 as *n* increases. Schneider (2016) provides a detailed overview of the different propositions underlying the Fibonacci sequence. The golden ratio and its variants have been applied in many ways in technical analysis, namely Fibonacci arcs, fans, and projections.^3^ Due to this study’s scope, we focus predominantly on Fibonacci retracements.

Fibonacci retracements can be utilized to complement another trading approach and serve as a standalone technique for identifying pullback entries, making them of practical significance. One of the most significant advantages of Fibonacci retracement is the automation of the retracement levels. These levels can be widely used in day and swing trading across all products, such as grains, stocks, forex, treasuries, and other commodities. The measurements are relative and adjustable to any market and time frame used. Due to its self-fulfilling prophecy feature, many institutional and retail traders view the Fibonacci tool as an essential skill set for technical analysis. The primary focus of Fibonacci retracement ought to be at the level of 38.2% and 61.8% (Williams 2012).

Fibonacci retracements are particularly relevant as a powerful tool to predict the future in the context of the stock markets, which registered its worst performance since 2008 during the COVID-19 pandemic. For example, during the 2008–2009 economic recessions, the S&P 500 index hit its lowest point (at 666). Subsequently, the trend had been on a long-term upward movement, with a peak value at 3393, before the coronavirus prompted a plunge in March 2020. Technical strategists suggest that if the 2009 low S&P 500 point of 666 is the bottom (i.e., 0%) and the 2020 high of 3393 is 100%, based on two Friday closes in a row, then the support level corresponds to 38.2% and ultimately, an index value of 2351. If the trend continues, then the 61.8% level will reach 1708.^4^ There was a drastic fall in gold prices during 2012–2015, when the price fluctuated between $1200 and $1400 until June 2019, after which an upward swing was observed. The S&P Information Technology index had made a 50% retracement of the slump between January 26 and February 08, 2018.

As reported by Schneider (2016), variations to the conjugate golden ratio lead to Fibonacci retracement levels, which are set at 23.6%, 38.2%, 61.8%, and 78.6%, and are formulated as follows:

4 $$\begin{aligned} {\text{Limits}} = {\text{ Retracement levels}} \\ & \left( {\begin{array}{*{20}c} {\mathop {\lim { }}\limits_{n \to \infty } \frac{{\theta_{n} }}{{\theta_{n + 3} }} \mathop {\lim }\limits_{n \to \infty } \frac{{\theta_{n} }}{{\theta_{n + 2} }} } \\ {\mathop {{\text{lim}}}\limits_{n \to \infty } \frac{{\theta_{n} }}{{ \theta_{n + 1} }} \mathop {{\text{lim}}}\limits_{n \to \infty } \sqrt {\frac{{\theta_{n} }}{{\theta_{n + 1} }}} } \\ \end{array} } \right) = \left( {\begin{array}{*{20}c} {23.6\% 38.2\% } \\ {61.8\% 78.6\% } \\ \end{array} } \right). \\ \end{aligned}$$

Nowakowski and Borowski (2005) provided in-depth details of further retracements and expansion levels, all from variations in the conjugate golden ratio. As Kumar (2014) outlined, these levels are imposed onto a stock price chart, following the identification of a swing high and a swing low over a specific period. Another standard retracement level used is 50%, in line with the Gann theory (see Gann 1949) in which prices are expected to retract by 50%. A swing high (low) occurs when the high (low) price reached is higher (lower) than a given number of highs (lows) positioned around it. When a swing low event follows a swing high event, the Fibonacci retracements levels can function as support at the different levels, with the time set between the two events. Similarly, the retracement levels can act as resistance when a swing high event follows a swing low event, with the time set between the two events. The different corresponding stock and crypto prices relative to each retracement level are calculated as follows:

5 $${\text{Swing\,low\,price }} + \left( {\begin{array}{*{20}c} {23.6\% } \\ {\begin{array}{*{20}c} {38.2\% } \\ {50\% } \\ {61.8\% } \\ {78.6\% } \\ \end{array} } \\ \end{array} } \right) \cdot \left| {\begin{array}{*{20}c} {\Delta } \\ \end{array} } \right|$$

6 $${\text{Swing\,high\,price }} - \left( {\begin{array}{*{20}c} {23.6\% } \\ {\begin{array}{*{20}c} {38.2\% } \\ {50\% } \\ {61.8\% } \\ {78.6\% } \\ \end{array} } \\ \end{array} } \right) \cdot \left| {\begin{array}{*{20}c} {\Delta } \\ \end{array} } \right|$$

where Δ is the absolute difference between the swing high and swing low prices, initially, we took those swing prices to be where trends change direction. Equation (5) applies for support levels; Eq. (6) is applicable for resistance levels.

### Price crossover strategy

We pursued a price crossover strategy in line with Gurrib (2016), who put together an optimized MA strategy, and Murphy (1999), who introduced double crossovers. In line with all MA, the broad length of the MA defines the timeframe for the trading system. A system using 26-day and 9-day SMAs is usually categorized as short-term. Similarly, a trading rule using a 100-day or 200-day SMA would be considered a medium-term or long-term strategy. A bullish price crossover occurs when the spot price crosses above the longer MA, commonly referred to as a golden cross. Conversely, a bearish crossover is observed when the spot price crosses below the longer MA, traditionally considered a dead cross. This study selected a 50-day MA. The price crossover trading strategy was set as follows:

7 $$\left( {\begin{array}{*{20}c} {\delta_{t - 1} \left\langle {SMA_{t - 1} , \delta_{t} } \right\rangle SMA_{t} } \\ {\delta_{t - 1} > SMA_{t - 1} , \delta_{t} < SMA_{t} } \\ \end{array} } \right) \to \left( {\begin{array}{*{20}c} {Golden cross} \\ {Dead cross} \\ \end{array} } \right) \to \left( {\begin{array}{*{20}c} {Buying signal} \\ {Selling signal} \\ \end{array} } \right)$$

### Setting up the trading strategy

Before testing whether Fibonacci retracements work in energy markets, it is crucial to examine their existence in uptrend or downtrend motions. Although different ways can be used to determine the presence of an uptrend or downtrend, this study calculated the slope of linear regression based on the daily closing prices. We chose a minimum of 50 days to allow the regression to capture enough movements in the energy prices without excessive unreliable up or downtrends. An area of future research could consider validating the slopes over different regression periods.

## Research findings

### Descriptive statistics

**Fig. 4:**
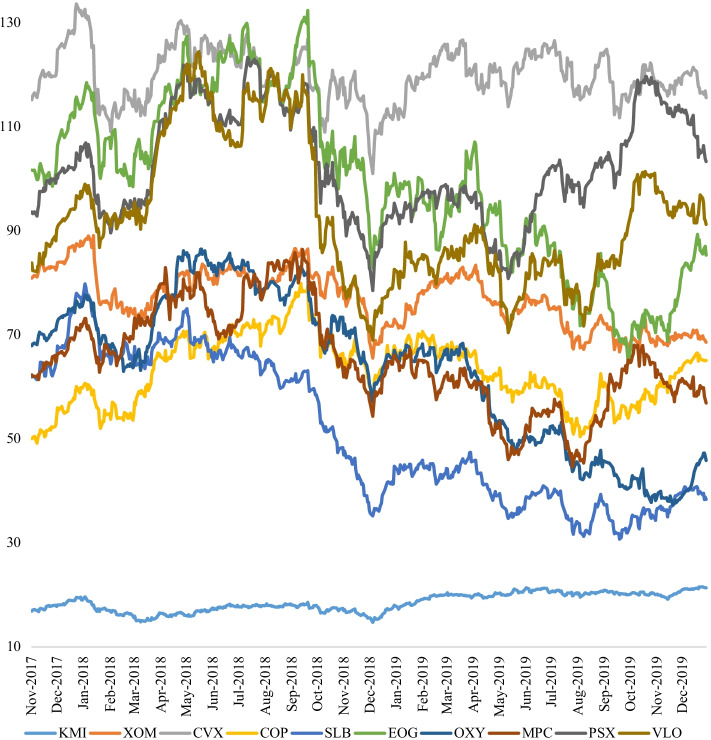
Leading U.S. energy stocks (Nov 2017–Jan 2020). *Note*: This figure reports the daily equity prices, at close, for the ten energy companies, which are all listed as leading stocks under the S&P 1500 Composite Energy index. The stocks (trading symbols) include Kinder Morgan Inc (KMI), Exxon Mobil (XOM), Chevron Corp (CVX), ConocoPhillips (COP), Schlumberger Ltd (SLB), EOG Resources (EOG), Occidental Petroleum (OXY), Marathon Petroleum Corp (MPC), Phillips 66 (PSX), and Valero Energy Corp (VLO). The data covers the period Nov 2017–Jan 2020. *Source*: S&P 500 Dow Jones Indices, Factset

Figure 4 displays the daily equity prices at close for the leading energy stocks of the S&P 1500 Composite Energy index. We captured 543 daily observations for each stock. As expected, their prices mainly behaved in the same fashion from November 2017 to January 2020. Correlation values varied from − 0.69 to 0.95 among the energy stocks. Following the exclusion of KMI, the correlation values ranged from 0.2 to 0.95. With values extending from $14.71 for KMI to $133 for CVX, the average stock prices stretched from $18.53 for KMI to $119.90 for CVX. While KMI had the smallest risk value with a standard deviation (SD) of $1.74, EOG had the highest risk with a value of $16.65. Half of the energy stocks were negatively skewed, with the remaining half (COP, SLB, MPC, PSX, VLO) exhibiting a positive skew. The skewness values ranged between − 0.5 and 0.5, signifying fairly symmetrical distributions. Except for CVX, which had a kurtosis value of nearly zero, the remaining energy stocks were platykurtic, with negative kurtosis values ranging from − 0.56 for MPC to − 1.52 for SLB. Although not reported here, correlation values among energy cryptos were significantly positive, extending from 0.79 to 0.94. The average prices ranged from $0.0189 for TSL to $0.3090 for GRID. Similarly, SD was the smallest (highest) for TSL (GRID). Distributions of energy crypto prices were positively skewed and leptokurtic.

### Trends in energy stock and crypto markets

**Fig. 5:**
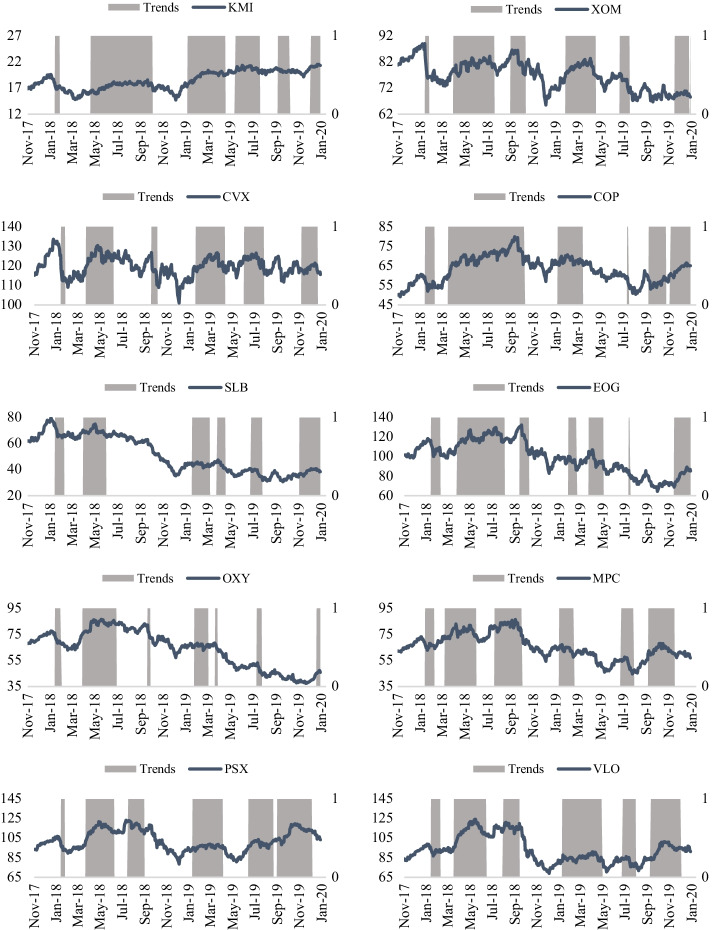
Energy stock prices and trends (Nov 2017–Jan 2020). *Note:* The company stocks (trading symbols) include Kinder Morgan Inc (KMI), Exxon Mobil (XOM), Chevron Corp (CVX), ConocoPhillips (COP), Schlumberger Ltd (SLB), EOG Resources (EOG), Occidental Petroleum (OXY), Marathon Petroleum Corp (MPC), Phillips 66 (PSX), and Valero Energy Corp (VLO). A trend is assumed to come into existence if the slope of the last 50 days is greater or less than zero. An upward (downtrend) trend is in place when the slope is positive (negative). The grey (white) areas are periods with uptrends (downtrends). *Source*: S&P 500 Dow Jones Indices, Coinmarketcap, and Factset

**Fig. 6:**
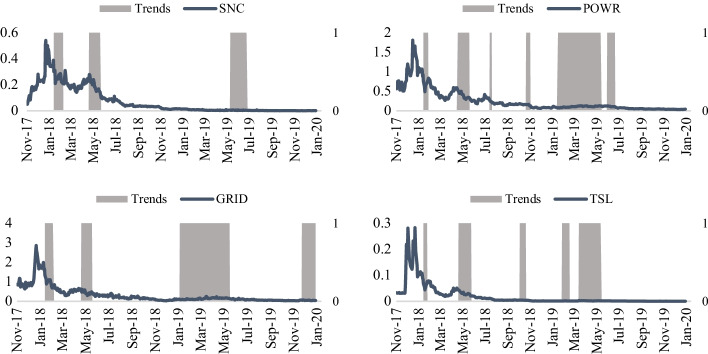
Energy crypto prices and trends (Nov 2017–Jan 2020). *Note*: The four energy cryptos are also listed, namely SunContract (SNC), Powerledger (POWR), Grid + (GRID) and Energo (TSL). A trend is assumed to come into existence if the slope of the last 50 days is greater or less than zero. An upward (downtrend) trend is in place when the slope is positive (negative). The grey (white) areas signify periods with uptrends (downtrends). *Source*: S&P 500 Dow Jones Indices, Coinmarketcap, and Factset

Figure 5 captures the relationship between different energy stock prices and their respective trends, and Fig. 6 captures the relationship between different energy crypto prices with their trends. A trend is assumed to come into existence if the slope of the last 50 days is greater or less than zero. An upward (downtrend) trend is in place when the slope is positive (negative). An upward (downtrend) trend continues until the slope turns negative (positive). In line with the price crossover strategy, this allows capturing enough movements in the energy prices without giving excessive unreliable up or downtrends. Other periods were also used in the slope value estimations, but the results were not improved. The gray areas represent the periods with uptrends, while the white spaces in between signify the downtrends. As observed from Fig. 5, the trends in the energy stock prices tend to be mostly in line with the ongoing prices. More importantly, trends tend to follow the same direction in most energy stock markets. For illustration, between April 2018 and June 2018, on average, all equity prices witnessed increases in an uptrend period. It is imperative to capture that each slope is based on a 50-day period calculation. Comparatively, for the energy cryptos (Fig. 6), the prices did not witness uptrends compared to energy stock prices. The lack of uptrends can be attributed to a more frequent downtrend in the energy crypto markets in late December 2017 or early January 2018, when crypto prices fell dramatically from their prior highs. While uptrends and downtrends are easily noticeable for energy stocks, a downtrend scenario is assumed for energy cryptos, starting from December 2017 or early January 2018, depending on the highs of each cryptocurrency around that time.

### Fibonacci retracements

**Fig. 7:**
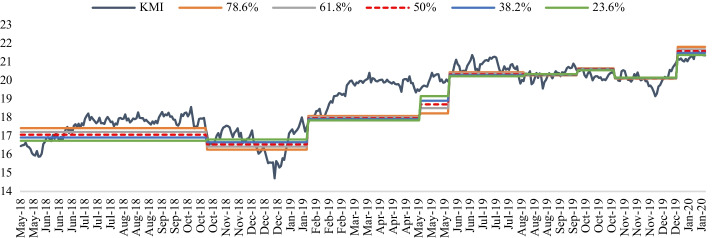
KMI retracement levels. *Note*: Fig. 5 reports the daily equity prices, at close, for Kinder Morgan Inc (KMI). The 23.6%, 38.2%, 50%, 61.8%, and 78.6% Fibonacci retracement levels were applied to KMI prices from November 2017 to January 2020. Swing high and swing low prices were taken as the prices at the start of the current and previous trends. *Source*: Factset and S&P 500 Dow Jones Indices

In line with Eqs. (5) and (6), the Fibonacci retracements were applied to the energy stocks and energy crypto prices from November 2017 to January 2020. The swing high and swing low prices were initially taken as the prices where new uptrends/downtrends would occur. However, this resulted in retracements ranges failing to capture most, or all, price movements in the next trend in place. For example, Fig. 7 shows how KMI retracement levels were not broad enough.

**Fig. 8:**
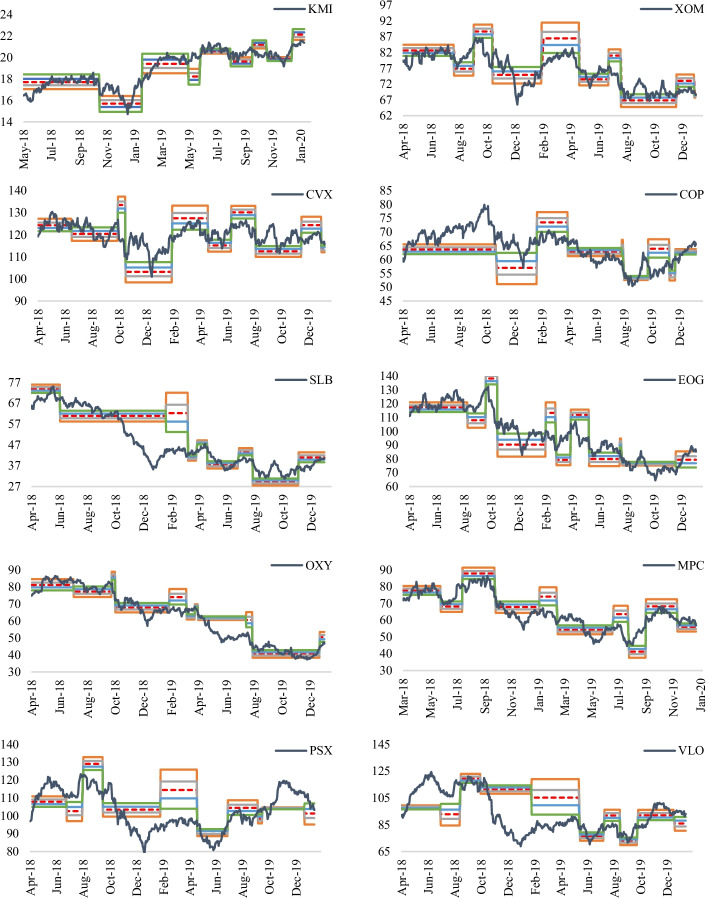
Energy stock prices and Fibonacci retracements. *Note*: This figure represents the 23.6%, 38.2%, 50%, 61.8% and 78.6% (the colour codes are provided in Fig. 7) Fibonacci retracement levels for the leading U.S. energy stock prices over the period November 2017–January 2020

**Fig. 9:**
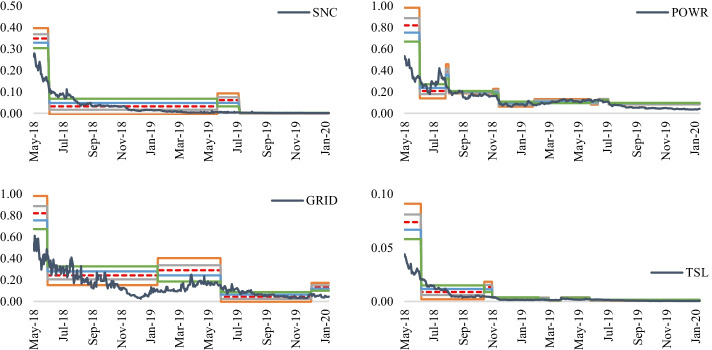
Energy crypto prices and Fibonacci retracements. *Note*: This figure represents the 23.6%, 38.2%, 50%, 61.8% and 78.6% (the colour codes are provided in Fig. 7) Fibonacci retracement levels for the leading U.S. energy crypto prices over the period November 2017–January 2020

Consequently, Eqs. (5) and (6) were updated, with swing high (low) prices representing the highest (lowest) prices within a specific period, and prices are either trending upwards or downwards. For instance, if the previous period had an uptrend, the difference between the highest and lowest prices is selected during that uptrend—Figs. 8 and 9 capture how the energy stock and energy crypto prices behave around Fibonacci retracements levels. The Fibonacci tool tends to capture price movements of energy stocks relatively better than energy cryptos. Despite the higher volatility found in cryptos, relative to energy stocks, the energy cryptos, like most major cryptos, such as Bitcoin, Ethereum, and Ripple, witnessed their highest peaks between November and December 2017. Comparatively, energy stocks fluctuated within more defined price ranges between November 2017 and January 2020, allowing tools, such as Fibonacci retracements, to better capture price movements. Noticeably, all the energy stocks prices mainly trended in the same fashion, with an uptrend for all stocks around April/May 2018. Similarly, around January 2019, all energy stocks witnessed price increases.

**Fig. 10:**
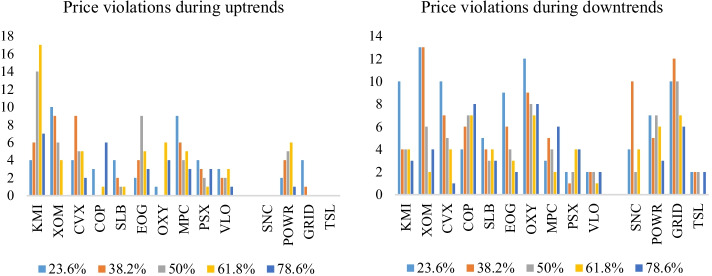
Price violations

While Fibonacci retracement levels tend to capture energy stock prices relatively well compared to energy crypto prices, it is worthwhile to analyze the existence of price violations during an uptrend or downtrend. Figure 10 displays the price violations which occurred against the five retracement levels. During an uptrend, energy cryptos witnessed the least price violations (SNC and TSL with no price violations); however, KMI accumulated the highest number of violations, with 48 violations at different support levels. XOM and MPC followed, with 29 and 27 support violations, respectively. Relatively, the number of price violations during a downtrend was higher than during uptrends. There were more violations for the ten energy stocks during downtrends than uptrends for seven of the stocks, except for KMI, MPC, and VLO. Energy cryptos followed the same trend, i.e., price violations of the retracement levels for all cryptos during periods of downtrends. More importantly, during uptrends, the highest number of violations occurred at the 61.8% retracement level. In contrast, during downtrends, numerous violations occurred at the 23.6% level. These findings suggest that while the Fibonacci retracement tool captured most of the downward movements in energy stock and crypto prices during an uptrend, price increases during downtrends were omitted. Noticeably, constituents with more price violations at a particular level of retracement tend to have price violations at other levels. This raises the critical question of whether violations during an uptrend (downtrend), e.g., at 61.8% (38.2%), are followed by violations at the prior retracement levels of 50% (23.6).

We looked back for up to 3 days to determine whether another price violation preceded price violations at a specific, different retracement level. We analyzed more than 1 day back to allow the energy stock and crypto prices to fluctuate and potentially cross retracement levels. For example, we investigated if a price violation during an uptrend or downtrend, e.g., the 50% retracement level (1 day, 2 days, and 3 days back), is followed by a price violation at the 38.2% level. The analysis was decomposed into uptrend and downtrend periods for both stocks and cryptos. Table 2 reports the existence of price violations, where a current price violation at a specific retracement level was preceded by another price violation at the prior descending or ascending retracement level. We analyzed violations at the 23.6%, 38.2%, 50%, and 61.8% levels, but not violations at 78.6%, as this is the upper boundary of our Fibonacci retracement levels. We assumed prices could not have broken a higher retracement level 1, 2, or 3 days back when the 78.6% level was currently broken. Most price violations (e.g., at time *t*) were preceded by price violations at the next higher retracement level at time *t-1*. This was more noticeable during downtrends, when retracement levels were broken more frequently 1 day before, including the current retracement break. There were fourteen instances when a 23.6% retracement level was broken for energy stocks, preceded by a 38.2% retracement 1 day before the 23.6% retracement break. Energy cryptos did not seem to witness consecutive violations in retracement levels, whether during an uptrend or downtrend. The highest number of consecutive price violations for energy cryptos occurred during downtrends. Only four retracement breaks occurred consecutively 1 and 2 days back, at the 50% and 61.8% levels.

**Table 2:** Behaviour of price violations

	Uptrend				Downtrend			
	23.6%	38.2%	50%	61.8%	23.6%	38.2%	50%	61.8%
*1 Day prior to current break*								
Energy stocks								
KMI	0	0	2	3	3	0	1	0
XOM	0	2	1	0	4	2	0	1
CVX	1	0	1	1	3	4	0	0
COP	0	0	0	0	0	0	2	0
SLB	0	0	0	0	1	0	0	0
EOG	0	0	0	0	0	1	1	1
OXY	0	0	0	1	3	1	1	0
MPC	4	0	1	1	0	2	1	0
PSX	0	0	0	1	0	0	0	0
VLO	1	1	0	0	0	0	0	0
Cryptos								
SNC	0	0	0	0	0	0	0	0
POWR	0	0	0	0	0	1	2	0
GRID	0	0	0	0	0	1	2	0
TSL	0	0	0	0	0	0	0	0
	23.6%	38.2%	50%	61.8%	23.6%	38.2%	50%	61.8%

	Uptrend				Downtrend			
	23.6%	38.2%	50%	61.8%	23.6%	38.2%	50%	61.8%
*2 Days prior to current break*								
Energy stocks								
KMI	0	0	4	0	0	1	0	1
XOM	3	0	0	0	1	1	2	1
CVX	0	2	0	0	2	0	1	0
COP	0	0	0	0	0	1	0	1
SLB	0	1	0	0	0	0	1	1
EOG	0	2	1	0	1	1	0	1
OXY	0	0	0	0	0	3	2	0
MPC	0	0	0	1	0	0	0	0
PSX	1	0	0	0	1	0	0	1
VLO	0	0	1	0	1	0	1	0
Cryptos								
SNC	0	0	0	0	0	0	0	0
POWR	0	0	0	0	0	0	0	2
GRID	0	0	0	0	0	0	0	2
TSL	0	0	0	0	0	0	0	0
	23.6%	38.2%	50%	61.8%	23.6%	38.2%	50%	61.8%

	Uptrend				Downtrend			
	23.6%	38.2%	50%	61.8%	23.6%	38.2%	50%	61.8%
*3 Days prior to current break*								
Energy stocks								
KMI	0	0	1	1	0	0	0	1
XOM	0	1	1	0	2	0	0	0
CVX	0	0	2	1	0	0	0	1
COP	0	0	0	0	0	1	1	1
SLB	0	0	0	0	0	0	1	1
EOG	0	0	1	0	0	1	0	0
OXY	0	0	0	1	0	0	0	2
MPC	1	0	1	0	0	0	0	1
PSX	1	1	0	0	0	0	0	0
VLO	1	1	0	0	0	0	0	0
Cryptos								
SNC	0	0	0	0	0	0	0	0
POWR	0	0	0	1	0	0	0	1
GRID	0	0	0	1	0	0	0	1
TSL	0	0	0	0	0	0	0	0
	23.6%	38.2%	50%	61.8%	23.6%	38.2%	50%	61.8%

As we moved from 1 to 2 and 3 days back, fewer consecutive retracement breaks occurred, suggesting that most retracement levels were broken consecutively within 1 day. Interestingly, most of the price violations for energy stocks, accompanied by a prior price violation 1, 2, or 3 days before, occurred at the higher retracement levels of 50% and 61.8%. This suggests that price violations tend to occur more frequently when the 61.8% and 50% are broken, with 78.6% and 61.8% preceding such price violations during a short period. In other words, the number of consecutive price violations at the 23.6% and 38.2% retracement levels was relatively lower than the 61.8% and 50% levels. During an uptrend, prices are expected to rise, following which price violations are likely to occur. This explains why the 50% and 61.8% retracement levels tend to be broken more consecutively than other lower retracement levels. Similarly, during downtrends, prices are expected to fall, after which price violations tend to occur. The only exception to this was during downtrends when most of the price violations took place consecutively at the lower retracement levels of 23.6% and 38.2%.

Based on the above findings in which retracements tend to witness lesser price violations at lower retracement levels, we put together a trading strategy to test the use of Fibonacci retracement levels on energy stock and crypto prices. During an uptrend, a long position was pursued when the price crossed over the 23.6% retracement level, and the position closed out when the price crossed under the 61.8% retracement level. Similarly, a short position was pursued during downtrends when the price crossed under the 23.6% level, with a subsequent long position after crossing over the 61.8% level. Table 3 provides a summary of the retracement levels.

**Table 3:** Summary of retracement levels

Position	Uptrend	Downtrend
Long	$Price_{t - 1} < 23.6\% retracement < Price_{t}$	$Price_{t - 1} < 61.8\% retracement < Price_{t}$
Short	$Price_{t} < 61.8\% retracement < Price_{t - 1}$	$Price_{t} < 23.6\% retracement < Price_{t - 1}$

Assuming that a transaction is based on the purchase or sale of one stock and that long or short energy stocks can be transacted without restrictions, like a buy (sell) followed by a sell (buy), the total net profit, or loss, during periods of uptrends and downtrends is calculated as follows:

8 $$Total\,return = \frac{{\mathop \sum \nolimits_{u}^{s} price + \mathop \sum \nolimits_{d}^{s} price + \varphi .n}}{{\mathop \sum \nolimits_{u}^{l} price + \mathop \sum \nolimits_{d}^{l} price + \theta .n}} - 1$$

where $\sum\nolimits_{u}^{s} p rice$ represents the sum of all prices in which short positions were taken during an uptrend; $\sum\nolimits_{d}^{s} p rice$ denotes the sum of all prices in which short positions were taken during a downtrend; $\sum\nolimits_{u}^{l} p rice$ and $\sum\nolimits_{d}^{l} {price}$ signify the sum of all prices in which long positions were taken during periods of uptrends and downtrends; $\varphi$ refers to the price at which open positions are closed at the end of the trading period, where open positions were net long before the close of all positions. Similarly, $\theta$ represents the price at which open positions are closed at the end of the trading period, where open positions were net short before the close of all positions; *n* is the number of open positions at the end of the trading period, just before they were offset with a close. Due to the approach taken to calculate the return, the average risk was proxied using the average SD of energy prices. All positions were closed at the end to allow for comparison with the buy-and-hold strategy. Buy-and-hold returns were based on a buy on November 28, 2017, and a subsequent sale on January 17, 2020.

As shown in Table 4, six of the ten energy stocks displayed long positions during uptrends, while only KMI, EOG, and OXY exhibited net short positions. Comparatively, eight of the energy stocks had net short positions during downtrends, except for COP and PSX. This suggests that during uptrends (downtrends), energy stocks tend to attract more buys (sales) based on traders’ use of the Fibonacci retracement strategy. Assuming that a transaction is based on the purchase or sale of one stock and that long or short energy stocks can be transacted without restrictions, like a buy (sell) followed by a sell (buy), we can calculate the total net profit or loss during uptrends and downtrends. Apart from KMI, XOM, CVX, and OXY, the remaining energy stocks reported positive total returns, ranging from 4% for SLB to 177% for COP. The negative performance of XOM and CVX can be attributed to their negative gains, particularly during uptrend periods when they reported $502.5 and $121.9 losses, respectively. The average risk ranged from $5.22 for KMI to $27.44 for CVX.

**Table 4:** Performance evaluation of Fibonacci-based strategy

	KMI	XOM	CVX	COP	SLB	EOG	OXY	MPC	PSX	VLO
*Panel A: Energy stocks*										
Net positions (uptrend)	− 11	6	0	4	3	− 1	− 5	4	6	3
Net positions (downtrend)	− 5	− 13	− 5	2	− 2	− 8	− 8	− 4	0	− 2
Total gain (uptrend)	216.20	− 502.5	− 121.9	− 202.1	− 124.57	511.06	493.47	− 197.6	− 514.8	− 253.1
Total gain (downtrend)	100.84	922.17	586.88	− 124.19	99.73	738.81	433.98	270.37	1.69	184.57
Total return	− 5%	− 4%	− 7%	177%	4%	44%	− 770%	12%	11%	4%
Average risk	5.22	20.44	27.42	12.60	9.20	22.30	16.44	15.46	19.37	17.62
Sharpe	− 0.014	− 0.003	− 0.003	0.139	0.002	0.019	− 0.470	0.006	0.004	0.001
Sharpe per trade	0	0	0	0.006	0	0.001	− 0.012	00	0	0
Buy-and-hold returns	25.4%	− 16.1%	− 0.7%	29.8%	− 38.4%	− 14.6%	− 33.5%	− 8.6%	9.8%	9.6%

	SNC	POWR	GRID	TSL
*Panel B: Energy cryptos*				
Net positions (uptrend)	0	− 3	4	0
Net positions (downtrend)	− 2	− 4	− 5	− 5
Total gain (uptrend)	0	0.37	− 0.81	0
Total gain (downtrend)	0.01	0.90	0.64	0.04
Total return	10%	66%	− 5%	1595%
Average risk	0	0.04	0.05	0
Sharpe	24.10	17.34	− 1.45	16,568.33
Sharpe per trade	2.01	0.54	− 0.06	1656.83
Buy-and-hold returns	− 0.986	− 0.938	− 0.960	− 0.987

Panel A of the Table 4 summarizes the performance evaluation values of investing in the top ten U.S. energy companies. Panel B summarizes the results of four energy cryptos. Average risk and average returns are based on arithmetic averages. Sharpe values captures the reward to volatility ratio. The U.S. 3-month Treasury bill rate was used as a proxy for the risk free asset. Buy and hold returns represent the returns for opening a position at the start and closing the position at the end of the trading period. Fibonacci retracement-based returns were calculated by closing any remaining open positions at the end of the period. Net positions are the number of short positions deducted from long positions. The period covered is Nov 2017–Jan 2020

Sharpe values were relatively low; the highest value was 0.139 for COP. This was consistent with the highest Sharpe per trade value of 0.006 for the same energy stock. Compared to the Fibonacci-based trading strategy, buy-and-hold returns reported negative returns for six of the energy stocks. The highest (lowest) return of nearly 29.8% (− 38.4%) was found in COP (SLB). For the energy cryptos, the use of our Fibonacci-based strategy resulted in very few trades. Only GRID reported net long positions during uptrends, while POWR reported net short positions, with the other two energy cryptos showcasing no transactions. During periods of downtrends, all four energy cryptos reported net short positions. All the cryptos had positive total returns except for GRID, which reported a loss of 5%. TSL had a very high total return relative to all stocks and cryptos, primarily because cryptos have only net short positions during downtrends. These open positions were all closed at the end of the studied trading horizon. The low amount and type of transactions (short or long) resulted in the abnormally high Sharpe value for TSL. Buy-and-hold returns were negative for all cryptos instead of the positive performance observed under the Fibonacci-based strategy for four stocks.

Table 4 shows the results of a trading strategy based solely on the use of Fibonacci retracements. However, it is interesting to examine whether complementing the Fibonacci tool with a price crossover strategy results in a superior trading model for the energy commodities. Table 5 provides the findings of a Fibonacci retracement strategy complemented with price crossover rules. Due to the addition of price crossover rules to the existing model, fewer trading opportunities are expected. During uptrends, energy stocks tend to display relatively more short net positions, with only XOM reporting one net long position. Similar to the model based on Fibonacci retracements, only KMI, EOG, and OXY, with the addition of VLO, reported net short positions during uptrends. Comparatively, five of the energy stocks had net long positions during downtrends, while EOG reported a net short position. This suggests that energy stocks tend to attract more sales (buys) during uptrends (downtrends), based on traders following a Fibonacci retracement strategy complemented with a price cross strategy. Assuming that a transaction is based on the purchase or sale of one stock and that long or short energy stocks can be transacted without restrictions, like a buy (sell) followed by a sell (buy), we can calculate the total net profit or loss during periods of uptrends and downtrends. Except for KMI, XOM, CVX, and SLB, all energy stocks reported positive total returns ranging from 4% for COP to 34% and 35% for EOG and OXY, respectively. While the negative performance of XOM can be attributed to losses during both uptrend and downtrend periods, the negative returns observed for KMI and CVX were due to the closure of the open positions at lower prices at the end of the trading horizon. The average risk ranged from $2.52 for KMI to $10.72 for EOG, and Sharpe values were relatively low, with the highest value being 0.044 for OXY. This was closely consistent with the second-highest Sharpe per trade value of 0.0074 for the same energy stock. Compared to the Fibonacci-based trading and the buy-and-hold strategy, the model that complemented both the Fibonacci and price crossover strategy did not result in superior total returns; no transaction occurred for SLB due to the latter strategy.

**Table 5:** Performance evaluation of Fibonacci and price crossover strategies

	KMI	XOM	CVX	COP	SLB	EOG	OXY	MPC	PSX	VLO
*Panel A: Energy stocks*										
Net positions (uptrend)	− 6	1	0	0	0	− 1	− 2	0	0	− 2
Net positions (downtrend)	0	1	0	2	0	− 1	1	1	1	0
Total gain (uptrend)	116.32	− 81.38	− 1.12	0.00	0.00	158.08	163.87	0	0	186.10
Total gain (downtrend)	0	− 75.92	0	− 125.00	0	96.86	− 64.83	− 54.21	− 90.83	0
Total return	− 9%	− 13%	− 1%	4%	−	34%	35%	5%	14%	2%
Average risk	2.52	6.33	10.21	5.32	0	10.72	7.40	3.81	6.66	7.75
Sharpe	− 0.044	− 0.023	− 0.003	0.004	−	0.030	0.044	0.008	0.018	0
Sharpe per trade	− 0.0037	− 0.0058	− 0.0014	0.0010	−	0.0051	0.0074	0.0039	0.0088	0
Buy-and-hold returns	0.254	− 0.161	− 0.007	0.298	− 0.38	− 0.146	− 0.335	− 0.086	0.098	0.096

	SNC	POWR	GRID	TSL
*Panel B: Energy cryptos*				
Net positions (uptrend)	0	0	0	0
Net positions (downtrend)	− 1	1	2	0
Total gain (uptrend)	0	0	0	0
Total gain (downtrend)	0	− 0.12	− 0.62	0
Total return	40%	− 64%	− 67%	−
Average risk	0.00	0.01	0.02	−
Sharpe	3273.55	− 109.96	− 29.63	−
Sharpe per trade	1636.77	− 54.983	− 4.939	−
Buy-and-hold returns	− 0.986	− 0.938	− 0.960	− 0.987

Panel A of Table 5 summarizes the performance evaluation results of investing in the top ten U.S. energy stocks of the S&P Composite 1500 Energy index based on a Fibonacci retracement strategy which is complemented with a price crossover strategy. Panel B reports the results of four energy cryptos. Average returns and average risk are based on arithmetic averages. Sharpe values represent the excess return per unit of total risk. The U.S. 3-month Treasury bill rate was used as a proxy for the risk-free asset. Buy and hold returns denote the returns for opening a position at the start and closure of the position at the end of the trading period. Fibonacci retracement based returns are calculated by closing any remaining open positions at the end of the period. Net positions are the number of short positions deducted from long positions. The price crossover strategy was based on a 50-day MA. The period covered was Nov 2017–Jan 2020

The Sharpe and Sharpe per trade ratios barely improved and mainly were too low to attract investors’ attention. Using our Fibonacci-based strategy in conjunction with the price crossover strategy resulted in even fewer or no trading signals for the energy cryptos. During uptrends, no energy cryptos reported net long positions. However, POWR and GRID reported net long positions during the downtrend periods, with SNC reporting a net short position. Only SNC reported a total return of 40%, based on the closure of the net short position at the end of the investment horizon. However, POWR and GRID reported 64% and 67% negative returns, caused primarily by closing positions at lower prices. The low amount and type of transactions (short or long) resulted in the abnormally high Sharpe value for energy cryptos. Buy-and-hold returns were negative for all cryptos compared to the Fibonacci-based strategy, which yielded positive returns only for one crypto. This suggests that using the Fibonacci retracement tool complemented with the price crossover strategy is not warranted, potentially due to the significant down trending periods since January 2018, which allowed for no position during relatively small pockets of eventual uptrends. This resulted in performance measures, such as the Sharpe or Sharpe per trade being less reliable, due to very few or zero transactions.

## Conclusion

This study investigates the use of Fibonacci retracements as a technical analysis tool, which the extant literature has not sufficiently documented, particularly regarding (i) its application on energy stocks and cryptos, (ii) its usage as a strategy when complemented with a price crossover strategy, and (iii) its performance relative to a buy-and-hold trading strategy. As such, with a focus on the Fibonacci retracements strategy, this study explores the performance of the top-ten energy stocks of the S&P 1500 Composite Energy Index and four energy cryptos from November 2017 to January 2020.

With positive correlations ranging from 0.2 to 0.95, most energy stocks trended in the same direction under the study period. The Fibonacci retracement tool tended to capture energy stock prices better than energy cryptos. A possible explanation resides in the fact that energy stock prices fluctuate within a more defined range, allowing the technical analysis tool to capture the price movements better. To refine the use of the Fibonacci tool, we applied the difference between the highest and lowest prices during a prior trend and used it for future price movements. Price violations tended to occur more during downtrends compared to uptrends for both energy stocks and cryptos. While most down movements were captured during uptrends, price increases during downtrends were largely omitted. Constituents with relatively more price violations at a particular retracement level also tended to have more price violations at other retracement levels. The highest number of consecutive price violations occurred during downtrends. Less consecutive retracement breaks took place as we moved from 1 day to 2 and 3 days prior. Price violations tended to occur more when the 61.8% and 50% levels were broken, with the 78.6% and 61.8% levels being recently violated before such events. This suggests that, as expected, prices will cross these upper levels (50% and 61.8%) before being broken, as opposed to just crossing the lower levels (23.6% and 31.8%). Most energy stocks reported positive total returns, ranging from 4% for SLB to 177% for COP. We found similar results for energy cryptos. However, the performance of the Fibonacci-based strategy resulted in low Sharpe and Sharpe per trade values, warranting investors’ attention. While superior to the buy-and-hold model, the Fibonacci-based trading strategy did not significantly improve when complemented with a price crossover strategy, resulting in fewer or no trades in most instances and consequently unimpressive Sharpe values.

The policy implications are mainly in terms of speculators’ role in international financial markets, particularly commodities and energy equity markets. This study’s overall results suggest that, despite significant drops in oil prices, speculators (traders) can implement profitable strategies using technical analysis indicators, like the Fibonacci retracement tool, with or without price crossover rules. More importantly, prices are expected to break the 50% and 61.8% levels after their adjustments, further contributing to volatility. This provides some insights to regulating agencies, like the Commodity Futures Trading Commission and the Securities Exchange Commission, that, despite the substantial fall in the prices of commodity markets, like oil, which affected energy-based entities, traders in those energy stocks still enjoyed significant profits. Although the issue of whether speculators destabilize prices is outside this study’s scope, the overall results advise that the pass-through of oil drops from commodity markets to lower energy equities affects oil companies through lower stock prices. Nevertheless, this does not necessarily mean losses for speculators skilled in using technical analysis indicators, like the Fibonacci retracement tool. Alternatively, in our study, financialization brought some benefits to commodity speculators with access to energy stocks. Future avenues of research are warranted in terms of frequency, which can be modified to a higher (e.g., intraday) and lower (e.g., weekly) frequency. More importantly, future studies need to assess the period that defines a trend.

## Data Availability

The datasets used and/or analysed during the current study are available from the corresponding author on reasonable request.

## Abbreviations

GDP: Gross Domestic Product
IEA: International Energy Agency
GHG: Greenhouse gas
EMH: Efficient Market Hypothesis
RSI: Relative strength index
MA: Moving average
S&P: Standard & Poor’s
ADX: Average Directional Index
DJIA: Dow Jones Industrial Average
SMA: Simple Moving Average
SPDR: Standard & Poor's Depositary Receipt
GSCI: Goldman Sachs Commodity Index
SD: Standard deviation
KMI: Kinder Morgan Inc
XOM: Exxon Mobil
CVX: Chevron Corp
COP: ConocoPhillips
SLB: Schlumberger Ltd
EOG: EOG Resources
OXY: Occidental Petroleum
MPC: Marathon Petroleum Corp
PSX: Phillips 66
VLO: Valero Energy Corp
SNC: SunContract
POWR: Powerledger
GRID: Grid +
TSL: Energo

## Footnotes

1 The study by Duan et al. (2018) proposes a new optimal model termed the “gray model” for forecasting Chinese equity indices by combining the features of the Fibonacci sequence and Elliott Wave Theory.

2 https://coinmarketcap.com/.

3 Fibonacci arcs, fans, and retracements are the popular methods used by traders. The role of Fibonacci arcs is to highlight potential support or reversal when prices pull back after an earlier advance. Fibonacci fans, constructed to confirm support levels or potential reversal zones, are also beneficial for measuring the speed of a trend's direction. Fibonacci retracement levels can be used to highlight support and resistance levels when prices retrace. Fibonacci extensions are applicable when stock prices reach new highs or lows without support or resistance levels (Bhandari 2014).

4 https://www.smithsonianmag.com/science-nature/fibonacci-sequence-stock-market-180974487/.
